# Prevalence of Sleep-Disordered Breathing in Children Referring for First Dental Examination. A Multicenter Cross-Sectional Study Using Pediatric Sleep Questionnaire

**DOI:** 10.3390/ijerph17228460

**Published:** 2020-11-16

**Authors:** Gabriele Di Carlo, Francesca Zara, Milena Rocchetti, Angelica Venturini, Antonio José Ortiz-Ruiz, Valeria Luzzi, Paolo Maria Cattaneo, Antonella Polimeni, Iole Vozza

**Affiliations:** 1 Department of Oral and Maxillo-Facial Sciences, Sapienza University of Rome, 00161 Rome, Italy; francescazara94@gmail.com (F.Z.); milenarock94@gmail.com (M.R.); angelicaventurini93@gmail.com (A.V.); valeria.luzzi@uniroma1.it (V.L.); antonella.polimeni@uniroma1.it (A.P.); iole.vozza@uniroma1.it (I.V.); 2 Department of Dentistry, Section of Orthodontics, Faculty of Health, Aarhus University, 8000 Aarhus, Denmark; paolo.cattaneo@dent.au.dk; 3 Department of Dermatology, Stomatology, Radiology and Physical Medicine, University of Murcia, 30008 Murcia, Spain; ajortiz@um.es

**Keywords:** child, prevalence, sleep apnea syndromes *, sleep apnea, obstructive *, surveys and questionnaires

## Abstract

*Background*: Sleep-related breathing disorders (SRDB) are a group of pathological conditions characterized by a dysfunction of the upper airways. The value of SRDB’s prevalence, in the pediatric population, ranges from 2 to 11% depending on the different methodologies used in measure and the difficulties in the diagnosis. The aim of this study was to assess the prevalence of SRDB using the Pediatric Sleep Questionnaire (PSQ). *Methods*: 668 patients were enrolled from the Department of Oral and Maxillo-Facial Sciences, Sapienza University of Rome, Italy and from the Unit of Integrated Pediatric Dentistry, University of Murcia, Spain. The questionnaires were administered to patients with no previous orthodontic and surgical treatment who attended on the first visit at the two units of pediatric dentistry. Data regarding general health status were extracted from the standard anamnestic module for first visit. Prevalence and logistic regression models were computed. *Results*: The ages ranged from 2 to 16 years old (average 7 years old). The prevalence of SRDB was 9.7% for the entire sample. The models showed a positive correlation between three variables (snoring, bad habits, and anxiety) and SRDB. *Conclusions*: The prevalence obtained demonstrates the relevance of sleep disorders in the pediatric population and highlights the central role of pediatric dentists in the earlier diagnosis of these disorders.

## 1. Introduction

Sleep-related breathing disorders (SRDB) are one of the six major categories of sleeping disorders which compromise mental and physical health (Third International Classification of Sleep Disorders, 2014) [1]. In children, the severity of symptoms can increase from primary snoring, breathing resistance syndrome, obstructive hypoventilation, and obstructive sleep apnea syndrome (OSAS) [2,3]. Risk factors and comorbidity associated to SRDB have been broadly discussed in literature: reduced retropharynx’s volume, hypertrophic lymphatic organs, allergic rhinitis, dentoskeletal anomalies, and obesity are usually defined as risk factors [4,5,6,7]. Furthermore, a chronic short sleep jointly with SRDB can also result in an endocrine disruption determining cardiovascular, cerebrovascular, and other chronic disease, such as nocturnal enuresis (NE) [8]. SRDB and NE are closely related by a bijective relationship, and they can adversely influence each other [9,10,11,12,13,14,15]. SRDB should be suspected when there are breathing alterations during the night. Modifications of breathing can appear in different ways, but snoring is the most common. However, the absence of snoring could make the diagnosis of SRDB extremely hard to be accomplished. This could be one of the factors leading to the tendency to not diagnose the pathology [2,16,17].

The gold standard for the diagnosis of SRDB is the polysomnography (PSG), which evaluates physiological parameter in relation with sleep and weakness [18]. However, the use of PSG is conditioned by relatively low accessibility and high cost. Indeed, the pediatric OSAS is not diagnosed and treated adequately, because there are not enough laboratories where PSG is available in respect of good economical compromise [19]. As mentioned above, SRDB is a group of underdiagnosed pathologies that requires an interval of 5–6 months from the first medical examination to the definitive diagnosis [20]. For this reason, several questionnaires were introduced in the clinical practice. Amongst them, the PSQ is the most suitable, producing a good reliability of results in the study of prevalence [21]. Even though in literature, there are several studies about prevalence of SRBD using PSQ questionnaires, they have showed variable values of it. In fact, the prevalence of SRDB in pediatric populations has been reported in a range of 2 to 11% [19,21,22,23,24,25]. This could be explained by the different methodologies and sampling methods adopted. The heterogeneity of prevalence could underestimate the magnitude of SRDB and create a tendency towards the underdiagnosis of the pathology [21].

The aim of this study is to assess the prevalence of sleep disordered breathing in a population referring for first dental examination to pediatric dental services. A further goal was to investigate a correlation between general health status recorded from the first visit anamnestic data and sleep-disordered breathing using pediatric sleep questionnaires.

## 2. Materials and Methods

### 2.1. Population

A cross-sectional investigation was conducted using the Pediatric Sleep Questionnaire (PSQ) designed by Chervin et al. (2000) [26]. Data were collected at the Unit of Pediatric Dentistry, Department of Oral and Maxillo-Facial Sciences, Sapienza University of Rome, Rome, Italy and the Unit of Integrated Pediatric Dentistry, University of Murcia, Spain. The corresponding validated version was adopted for Spain and Italy, accordingly [27,28]. The age range was determined by the research team and was meant to be inclusive of the typical pediatric population who refers for dental as well as orthodontics pediatric first consultation.

### 2.2. Study Design and Sampling

The questionnaires were administered to patients, according to the following criteria: patients who received orthodontic or surgical procedure on soft and hard tissue before the first dental examination were excluded. The pediatric sleep questionnaires were applied without altering the original structure [29]. PSQ is constituted by 22 questions, which evaluate four categories of symptoms: snoring, extreme daily tiredness, hyperactivity, and obstacle of attention. All questions are simple and concise to support the compilation. It was possible to answer “yes” (1 point in the scale of evaluation), or “no/I do not know” (0 points in the scale of evaluation). In the questionnaire, there were also enquiries concerning school performance/hyperactivity (taken from category A of Diagnostic and Statistical Manual of Mental Disorders (DSM) IV): the answers esteeming the null or lack of attention were evaluated as “no” (0 points in the scale of evaluation) or considered “yes” (1 point in the scale evaluation) when the attention is decent or sufficient. For each patient, the total score was calculated. According to Chervin et al. [26], a score of 0.33 (corresponding to a score equal to 8) classified 85.7% of subjects with a sensibility of 0.83 and specificity of 0.87 as at risk of SRDB.

As part of standard procedures for first dental examination, anamnestic questions regarding general health status were administered. These data were considered to identify possible links to SRDB in our population. When evaluating different pathological conditions, for example hyperactivity or bronchial asthma, in case of positive response, additional data (i.e., a medical written diagnosis) were required to confirm the diagnosis.

### 2.3. Statistical Analysis

The total prevalence was computed as well as the partial prevalence recorder in Italy and Spain. All responses collected from the Italian and Spanish samples were examined with a logistic and quantile regression depending on the single variables. The statistical model of the logistic regression was used to study the correlation between a dichotomous variable of response and several explicative variables (quantitative and categorized). Consequently, different methods were used to esteem the goodness of fit index of the reduced model: Hosmer–Lemeshow’s test of adaptation and the receiver operating characteristic (ROC), [30,31]. Additionally, the quantile regression was adopted to esteem the variation between 75th and 95th percentile score evaluating the possible modification between demographic variables and general health information collected using the questionnaire abovementioned.

### 2.4. Ethical Approval

The study protocol complied with the Guidelines for Good Clinical Practice, according to the Declaration of Helsinki (1975). The legal guardian of each patient signed an appropriate consent form and filled an additional health history questionnaire out. The study was approved by the Council of the Department of Oral and Maxillo-Facial Sciences, Sapienza University of Rome, Rome, Italy (Prot. Number 42/2020-0000935) and by the Research Ethics Committee of the University of Murcia, Spain (Prot. Number 2936/2020).

## 3. Results

### 3.1. Dimensions of Sample

Data were collected from 668 patients, 447 from Department of Oral and Maxillo-Facial Sciences, Sapienza University of Rome, Italy and 221 from the Unit of Integrated Pediatric Dentistry, University of Murcia, Spain. The age ranged from 2 to 16 years old (average 7 years old). Three hundred and twenty-three were females, 328 males, and 17 did not report the name of the child, hence the gender is not available. The computation of prevalence was done firstly for Italian and Spanish population separately and secondly putting together both (Table 1).

#### Logistic Model

Data collected using the logistic model show that gender, anxiety, and snoring are statistically significant (Table 2).

The odds ratio of OSAS (in aerage and same conditions) is shown below:

- It is 2.03 higher amongst male than female group.
- It is 10.20 higher amongst children with snoring compared to who do not suffer from this problem.
- It is 3.97 higher amongst children with anxiety compared to those who do not have it.

## 4. Discussion

This study aims to know the SRBD prevalence in a pediatric population from Italy and Spain using the PSQ questionnaire [29]. This purpose comes up by the different range of prevalence present in literature originating from various methodologies of study used and from the difficulty in the diagnosis. The adoptions of different questionnaires in the literature is impairing comparability between studies. Indeed, these questionnaires have widely varying information regarding validity and reliability [19]. We adopted the PSQ questionnaire, which is, as scientifically verified, a valid and reliable tool that can be used to identify SRBDs in clinical research [26]. The total value of prevalence falls within the range claimed by different authors [19,21,22,23,24,25]. It is important to highlight that most studies on prevalence performed diagnostic testing on children with snoring. It has been shown that not all the patients with SRDBS are snorers. This could be one of the reasons why sleep apnea in children is underestimated [19]. On the contrary, our study assessed the prevalence considering patients with and without snoring. Our sample showed that the odds ratio for Obstructive Sleep Apnea Syndrome is two times higher than among male and female group. This is consistent with the findings of Lumeng and Chervin, whose concluded that the prevalence of SRDB does differ by sex with males being affected at rates comprised between 50 to 100% [19]. It is interesting to note that most previous studies that did not find sex difference in SRDB in children are not considering cohorts aged 13 years old or older. This suggests that puberty-related hormonal physiologic changes may enhance the effects of sex on SRDB prevalence among children [19].

Understanding a valid correlation between SRDB and other diseases is the first step to a timely diagnosis and a correct therapeutic approach. Indeed, the morbidity associated with SRDB is only beginning to be understood [19].

Our data show that a positive correlation between SRDB and snoring exists. Snoring is the most important nocturnal symptom that indicates the early presence of SRDB in children. The results of our study show a positive correlation between snoring and SRDB. Therefore, the risk of developing SRDB in the presence of snoring was four times more frequent. This is consistent with Baidas et al., who enrolled in his study 1600 children from 16 different schools in Saudi Arabia: they observed that the risk of developing SRDB was four times more frequent in children with snoring [32]. A Chinese study that recruited 11,420 children, ages 3 to 14, reported that the risk of developing SRDB was higher in patients with snoring. Another Japanese survey conducted in 25,211 subjects (6–15 years old) demonstrated a direct correlation between snoring and SRDB and concluded that snoring can be used as a clinical marker for an early diagnosis [33,34].

Nevertheless, most of the data present in literature reveal that just under 50% of children suffer from SRDB and snoring at the same time [35]. The correlation with snoring is one face of SRDB but per se in not enough to detect it. Perhaps the correlation between SRDB and anxiety is even more controversial topic than the association with snoring. In fact, while for some authors, SRDB can influence the mood of children causing anxiety, depression, and sometimes, a worsening of the quality of life; for others, the correlation is not too clear [36,37]. Our results show that a patient with anxiety had a four times greater risk of presenting a SRDB disease. Cortese et al., in a clinical trial with a sample of children between 5 and 7 years old, described a positive correlation between SRDB and neurobehavioral changes such as depression, anxiety, and attention disorders; Rosen et al. showed that there is a greater probability of developing behavior problems in patients with SRDB [38,39]. Therefore, an early diagnosis of SRDB can prevent or avoid an aggravation of anxiety, depression, and hyperactivity that can sometimes evolve into serious mental pathologies [40]. The different results described in the studies could derive from the variety of methodologies used to diagnose SRDB and the difficulty in diagnosing a mental problem in children, together with the possibility that behavioral problems are considered as comorbidities and not as diseases directly correlated with OSAS [38,39,40,41]. This difficulty in evaluation has been highlighted in recent studies, which indicate the need for more clinical trials to better clarify a possible link between SRDB and different comorbidities [41,42,43].

## 5. Conclusions

The prevalence obtained demonstrates the relevance of SRDB and their comorbidity in the pediatric population. Likewise, stands out the usefulness of the PSQ and the central role of the pediatric dentist in the early diagnosis of these disorders.

## Figures and Tables

**Table 1:** Prevalence of sleep-related breathing disorders (SRDB) in the sample.

Country	Prevalence (%)	Number of Patients with Score ≥8	Total Number of Patients
Italy	7.87%	35	447
Spain	13.57%	30	221
Italy and Spain	9.73%	65	668

**Table 2:** Logistic model.

Parameter	Estimate	Std. Error	Z-Value	Pr (>|z|)
Intercept	−3.51	6.47	−5.43	0.00
Age (calculated in months)	−0.06	0.04	−1.40	0.16
Gender	7.24	3.41	2.12	0.03 *
Natural childbirth	3.04	3.66	0.83	0.40
Breastfeeding	0.441	4.23	0.11	0.92
Bad habits	0.61	3.61	0.17	0.87
Anxiety	1.38	7.59	1.82	0.07 **
Asthma	3.86	5.69	0.67	0.50
Snoring	2.32	3.35	6.93	0.00 *
Fainting	4.35	1.00	0.43	0.67

Values at the ESTIMATE and STANDARD ERROR are intended times 10^−1^. * Data are statistically significant at * *p* value < 0.05, and a ** *p* value < 0.1 (Dispersion parameter for binomial family taken to be 1). Null deviance: 352.14 on 544 degrees of freedom. Residual deviance: 271.45.12 on 529 degrees of freedom (123 observations deleted due to missingness). AIC: 303.45. Number of Fisher Scoring iterations: 15. Controlling variables: kidney, thyroid.
